# Clinical features of MEN1 in children, adolescents, and young adults: a single-center study

**DOI:** 10.3389/fendo.2026.1886831

**Published:** 2026-08-17

**Authors:** Simone Della Valentina, Laura Pierotti, Elena Pardi, Chiara Sardella, Anna Dal Lago, Angela Michelucci, Maria Adelaide Caligo, Gabriele Materazzi, Gianluca Frustaci, Emanuele Federico Kauffmann, Ugo Boggi, Paolo Piaggi, Claudio Marcocci, Filomena Cetani

**Affiliations:** 1 Department of Clinical and Experimental Medicine, University of Pisa, Pisa, Italy; 2 Endocrine Unit, University Hospital of Pisa, Pisa, Italy; 3 Laboratory of Molecular Genetics, University Hospital of Pisa, Pisa, Italy; 4 Endocrine Surgery Unit, Department of Surgical, Medical and Molecular Pathology and Critical Area, University Hospital of Pisa, Pisa, Italy; 5 Division of General and Transplant Surgery, University of Pisa, Pisa, Italy; 6 Department of Information Engineering, University of Pisa, Pisa, Italy; 7 National Institute of Diabetes and Digestive and Kidney Diseases (NIDDK), National Institutes of Health, Phoenix, AZ, United States

**Keywords:** disorders of calcium/phosphate metabolism, GEP-NET, MEN1, MEN1 gene, PHPT, adolescent, young adult

## Abstract

**Background:**

Multiple endocrine neoplasia type 1 (MEN1) can present during childhood and adolescence, yet data on the full endocrine and non-endocrine phenotype in young patients remain limited.

**Methods:**

We conducted a retrospective single-center study of genetically confirmed MEN1 patients diagnosed at ≤21 years and followed between 2001 and 2024. Clinical features, surveillance findings, treatments, and outcomes were extracted from medical records.

**Results:**

The cohort included 21 patients (52% males), with a median age at genetic diagnosis of 15 years and a median follow-up of 9 years. At first evaluation, 33% were asymptomatic *MEN1* pathogenic/likely pathogenic variant carriers. During follow-up, primary hyperparathyroidism (PHPT) developed in 71% (median age 18 years) and was frequently complicated by renal and skeletal involvement (hypercalciuria, silent nephrolithiasis, and reduced bone mass). Parathyroidectomy was performed in 33% of patients with PHPT, with variable surgical approaches. Both postoperative hypoparathyroidism and persistent or recurrent disease were documented. Gastroenteropancreatic neuroendocrine tumors (GEP-NETs) occurred in 48% (median age ~19.5 years), predominantly non-functioning and pancreatic, with surgery in <40% and no metastatic disease. Pituitary adenomas were detected in 52% (mostly non-functioning); one pediatric prolactinoma responded to cabergoline. Adrenal involvement (14%), cutaneous lesions (angiofibromas 33%; lipomas 19%), and thoracic neuroendocrine lesions (19%, all >21 years) broadened the early-life MEN1 phenotype.

**Conclusions:**

In MEN1 patients diagnosed at ≤21 years, early disease expression is common and clinically meaningful. PHPT represents the earliest and most prevalent manifestation and is frequently associated with renal and skeletal morbidity despite sometimes mild biochemical abnormalities. GEP-NETs and pituitary adenomas are also prevalent by late adolescence/young adulthood and are often indolent, suggesting structured, age-adapted multidisciplinary surveillance and early cascade genetic testing in at-risk families.

## Introduction

Multiple Endocrine Neoplasia Type 1 (MEN1, OMIM 131100) is a rare genetic syndrome characterized by the development of endocrine tumors and non-endocrine manifestations, with high penetrance. The classic triad of MEN1 includes primary hyperparathyroidism (PHPT), neuroendocrine tumors of the gastrointestinal tract and pancreas (GEP-NET), and pituitary adenomas (PitNET), with prevalence of approximately ~100%, 40-90%, and 40%, respectively during the lifetime (1). The estimated prevalence of MEN1 in the general population is between 1 and 3 cases per 100,000 individuals (2–6).

Ninety per cent of patients carry a germline pathogenic/likely pathogenic (P/LP) variant in the *MEN1* gene, located on chromosome 11q13, which is responsible for the expression of the protein menin, a tumor suppressor of 610 amino acids. Menin interacts directly or indirectly with more than 50 different proteins involved in cellular processes such as cell adhesion, cell division, DNA repair mechanisms, and cell cycle progression (7). Therefore, menin serves a pattern of proteins involved in different cellular functions, regulating cellular processes. The P/LP variant in the *MEN1* gene corresponds to the first hit that is passed on to offspring by the autosomal dominant pathway. However, a second hit on the non-mutated allele is necessary for the disease to develop, which, as described in Knudson’s model, results in the loss of heterozygosity (LOH), leading to the complete loss of the oncosuppressive action of the menin protein (7).

The diagnosis of MEN1 is based on clinical and/or genetic criteria. A clinical diagnosis is established in (I) individuals with at least two major MEN1-associated manifestations; (II) one MEN1-related tumor and a first-degree relative with MEN1, or (III) a confirmed germline *MEN1* P/LP variant. Interestingly, about 10% of MEN1 patients lack an identifiable *MEN1* pathogenic variant and are classified as phenocopies. These individuals typically exhibit a milder disease course with a life expectancy similar to that of the general population. However, the absence of a *MEN1* deleterious variant does not exclude a genetic basis for the disease (8).

In the pediatric and adolescent population, the frequency and severity of MEN1-related manifestations vary due to factors such as age at screening initiation and heterogeneity of clinical presentation. The previous and the recent guidelines recommend early screening for neuroendocrine tumors, allowing for early diagnosis and treatment planning to manage life-threatening complications effectively (4, 6). However, lifelong surveillance initiated during childhood may also have important psychosocial implications. Studies in adults have consistently demonstrated increased disease-related concerns and impaired health-related quality of life (9–11). In contrast, recent data suggest that children and adolescents with MEN1 either affected or healthy carriers generally maintain a quality of life comparable to that of siblings without MEN1 and healthy peers. However, a sub analysis performed only in affected individuals with MEN1-related manifestations showed that they have a poorer physical, social, and school functioning, highlighting the impact of early disease expression on daily life and well-being (12). These findings underscore the need for a more comprehensive characterization of MEN1 manifestations during childhood, adolescence, and young adulthood and their clinical consequences (12).

The current literature includes only a limited number of studies specifically addressing the clinical presentation of MEN1 manifestations during childhood, adolescence and youth. These studies differ significantly in terms of age cut-offs, ranging from under 18 years to 31 years, and in sample sizes, which varies from 8 to 160 patients. Most of the available data focus primarily on the classic MEN1-associated manifestations, such as PHPT, PitNET, and GEP-NET. Consequently, there is a notable lack of information regarding adrenal involvement and non-endocrine manifestations in younger MEN1 patients, leaving important aspects of the disease spectrum in this age group underexplored (13–18).

The objective of this study was to assess the prevalence of endocrine and non-endocrine manifestations in MEN1 patients diagnosed at ≤21 years of age, as well as their clinical presentation and treatment outcomes.

## Materials and methods

This is a retrospective monocentric study on a cohort of patients with familial and sporadic MEN1 syndrome who were consecutively followed up at our outpatient clinic from January 2001 to December 2024. Data on the medical history of MEN1 manifestations were retrieved from our electronic patient records and analyzed anonymously.

Patients were eligible if the genetic diagnosis of MEN1 was established at ≤21 years of age. This cut-off was selected to document the transition from adolescence to young adulthood (19) and because the available studies are characterized by substantial heterogeneity in the age limits adopted, with no clearly established cut-off for defining pediatric and young MEN1 populations. Collected data included age at genetic diagnosis, *MEN1* variant type, presence or absence of symptoms and age at symptom onset, and the type of MEN1-related manifestations.

### Screening, diagnosis and follow-up

Patients underwent genetic testing either as part of familial screening following the identification of MEN1 syndrome in a first-degree relative or because of clinical suspicion of MEN1, particularly in the presence of early-onset PHPT. All patients underwent biochemical and instrumental evaluation according to previous guidelines beginning at 5 years of age (4, 20). During the study period, the surveillance framework remained largely unchanged, although the timing of certain imaging examinations, particularly for GEP-NETs and radiological tools available have evolved over time.

Annual tests for ionized calcium and parathormone (PTH) were measured starting at 8 years of age. The diagnosis of PHPT was defined as the presence of elevated serum calcium and elevated or inappropriately normal PTH concentration on two different occasions. For patients with PHPT, biochemical data, as well as the presence and type of disease complications, were collected at diagnosis and at the last follow-up. Bone mineral density (BMD) and Z-score were evaluated by DXA at diagnosis and at the last follow-up. The type of treatment performed for PHPT (surveillance, surgical or medical) was also recorded.

Annual biochemical screening included glucose, and insulin measurements from 5 years of age and gastrin, chromogranin A, glucagon after 10 years. Screening for GEP-NETs was performed by abdominal gadolinium-enhanced MRI, with the age at initiation based on the recommendations in force during the study period. MRI was generally preferred over CT to reduce exposure to ionizing radiation. GEP-NETs were diagnosed based on lesions with consistent radiological features identified on two consecutive examinations. Abdominal gadolinium-enhanced MRI was also used during follow-up according to the guidelines (4). Insulinoma was diagnosed according to Whipple’s triad and a positive fasting test, whereas non-functioning NETs were defined by the absence of hormone hypersecretion. Data collected included age at diagnosis, affected organ, number of lesions and size of the largest lesion at diagnosis and last follow-up, treatment approach, and, in surgically treated patients, histology, TNM stage, and metastatic status.

Serum prolactin and IGF-1 were measured annually from 5 years of age onward, and pituitary MRI was performed every 2 to 3 years (4). A functioning PitNET was suspected based on abnormal pituitary hormone levels and confirmed by the detection of a pituitary lesion on MRI. Prolactinoma was diagnosed in the presence of elevated prolactin levels after exclusion of macroprolactin interference. Conversely, a non-functioning PitNET was diagnosed in the presence of normal pituitary hormone levels and a pituitary lesion on MRI. Data collected included age at diagnosis, functional status, presence of local compressive symptoms, lesion size at diagnosis and at the last follow-up, and type of treatment received.

Regarding the localization of neuroendocrine lesions in the lungs/bronchi and thymus, a contrast-enhanced chest CT scan was performed starting at the age of 15, in accordance with previous guidelines (4).

The diagnosis of adrenal adenoma or hyperplasia was established based on radiological findings obtained by abdominal ultrasonography, MRI and CT scans. The same imaging modalities were used during follow-up according to the guidelines (4). Adrenal functional assessment was performed by measuring aldosterone and plasma renin concentrations, 24-hour urinary metanephrines and normetanephrines, and serum cortisol at 8.00 in the morning after 1 mg of dexamethasone. The age at diagnosis, type of lesion (adenoma or adrenal hyperplasia), functioning nature and size of the lesion/s at diagnosis and follow-up were collected.

Finally, in all patients, the presence of non-endocrine manifestations and their age of onset were collected. All patients underwent a complete examination of the skin at the time of diagnosis and at subsequent follow-up with the aim of identifying the presence of MEN1-related skin manifestations (angiofibromas, collagenomas, and lipomas). In the presence of cutaneous manifestation, the site, size and age of onset were defined.

### Assays

Blood samples were obtained between 8 and 9 a.m. after an overnight fast. Serum levels of albumin, calcium, creatinine, phosphate, magnesium, glucose, as well as 24-hour urinary excretion of calcium were assessed using standard laboratory methods.

Ionized calcium concentrations were determined using the ion-selective electrode method (Nova 8 Calcium Analyzer, Nova Biomedical). Serum PTH was measured using a second-generation chemiluminescence assay (DiaSorin) until October 2014, when it was replaced by a third-generation chemiluminescence assay (DiaSorin LIAISON). Serum 25-hydroxyvitamin D (25(OH)D) concentrations were measured using a radioimmunoassay (DiaSorin) until September 2013, followed by a chemiluminescence assay (IDS-iSYS, IDS plc) thereafter. The concentrations of bone-specific alkaline phosphatase (BSAP) were obtained using an immunoenzymatic assay (OCTEIA Ostase BAP; IDS Ltd., Boldon, Tyne & Wear, UK); serum N-MID osteocalcin (IDS Ltd., Boldon, UK), and S-CTX (Nordic Bioscience Diagnostics A/S, Herlev, Denmark) by ELISA. The age-specific reference ranges for SBAP and S-CTX are reported in **Supplementary Material** while previously published data were used for osteocalcin (21).

The estimated glomerular filtration rate (eGFR) was calculated using the Chronic Kidney Disease Epidemiology Collaboration equation for patients older than 18 years. In individuals under 18 years, the revised Schwartz Equation was used to estimate the GFR (22).

### Bone densitometry

BMD was measured using a Hologic QDR-4500 densitometer (Hologic Inc., Waltham, MA, USA), with BMD measurements performed at the lumbar spine (L1–L4, posterior-anterior projection), femoral neck, total femur, and one-third distal radius. The coefficients of variation for these measurements were 1.1% at the lumbar spine, 1.2% at the femoral neck, and 1.4% at 1/3 distal radius. Z-scores were used to interpret BMD values. The definition of reduced bone mass was based on Z-score values at least one of the three sites analyzed, with values below –2.0 standard deviations (SD) considered indicative of low bone mass, in accordance with the 2019 Pediatric Official Positions of the International Society for Clinical Densitometry. In patients aged <20 years, Z-scores were derived using a dedicated pediatric DXA Z-score calculator (23). The assessment of bone fractures was conducted using vertebral fracture assessment (VFA) as part of the densitometric examination as previously described (24).

### Gene nucleotide sequence analysis

Genomic DNA was extracted from index patients’ peripheral leucocytes with Qiagen DNeasy blood kit according to standard protocols (Qiagen, Venlo, The Netherlands), as previously described (25). Briefly, the entire coding region and intron/exon boundaries of *MEN1* (GenBank entry NM_001370259.2), *CDKN1B* (NM_004064.5), and *AIP* (NM_003977.4) genes were investigated by sequencing germline DNA from all index patients up to July 2017 followed by Next Generation Sequencing (NGS) analysis. NGS was performed using a custom-designed Agilent SureSelect QXT target sample library preparation and capture method (Agilent Technologies, USA). The panel contains coding exons and flanking regions of 9 genes associated with hyperparathyroidism: *MEN1* (NM_130803), *CDKN1A* (NM_001220777), *CDKN1B* (NM_004064), *CDKN2B* (NM_004936), *CDKN2C* (NM_078626), *CDC73* (NM_024529), *GCM2* (NM_004752), *CASR* (NM_001178065), *AIP* (NM_003977).

PCR-amplified DNA was sequenced in forward and reverse directions by direct cycle sequencing using BigDye Sequencing Reaction kit v.1.1 (Applied Biosystems, Foster City, CA, USA) and run-on ABI 3130XL automated sequencer (Applied Biosystems). Whenever a *MEN1* variant was identified in the index case, the mutational analysis of the region of interest was extended to first-degree relatives.

Sequence variants were described according to Human Genome Variation Society (HGVS) nomenclature and annotated with the corresponding dbSNP reference SNP (rs) identifier, when available. Variant pathogenicity was classified according to the American College of Medical Genetics and Genomics/Association for Molecular Pathology (ACMG/AMP) recommendations as pathogenic, likely pathogenic, variant of uncertain significance (VUS), likely benign, or benign. The ACMG/AMP evidence criteria supporting each classification (e.g., PVS1, PS1, PM2, PP3) were also reported.

### Multiplex ligation-dependent probe amplification assay

MLPA analysis was conducted on index patients whose NGS results were negative, to detect large monoallelic deletions or amplifications within the *MEN1*, *CDC73* and *CDKN1B* genes. The analysis was performed using the SALSA MLPA probemix kit P244-C1 (MRC Holland, Amsterdam, The Netherlands), following the manufacturer’s protocol as previously described (25). Each MLPA experiment included at least three reference DNA samples derived from healthy individuals with no expected copy number variations in the target regions. Additionally, a negative control sample (without DNA) and appropriate positive controls were included to ensure assay validity.

### Statistical analysis

Statistical analyses were performed using R software version 4.5.2. Data are presented as mean ± SD for normally distributed variables and as median value [interquartile range (IQR): Q1–Q3] for non-normally distributed variables. Categorical variables are expressed as number (percentage). All statistical tests were two-tailed, and a p value < 0.05 (α = 0.05) was considered statistically significant. Normality of continuous variables was assessed using the Shapiro–Wilk test. For between-group comparisons, Student’s *t*-test for independent samples was applied when data met normality assumptions otherwise, the Mann–Whitney U test was used. For within-subject comparisons (baseline vs follow-up), the paired *t*-test was used for normally distributed paired differences, whereas the Wilcoxon signed-rank test was applied when normality was not satisfied. Categorical variables were compared using the Fisher’s exact test.

## Results

### Clinical characteristics

The study cohort comprised 21 *MEN1*-positive patients, including 11 males (52%) and 10 females (48%), with a median age at genetic diagnosis of 15 years (IQR 9-19), in 14 distinct families, all unrelated.

**Figure 1:**
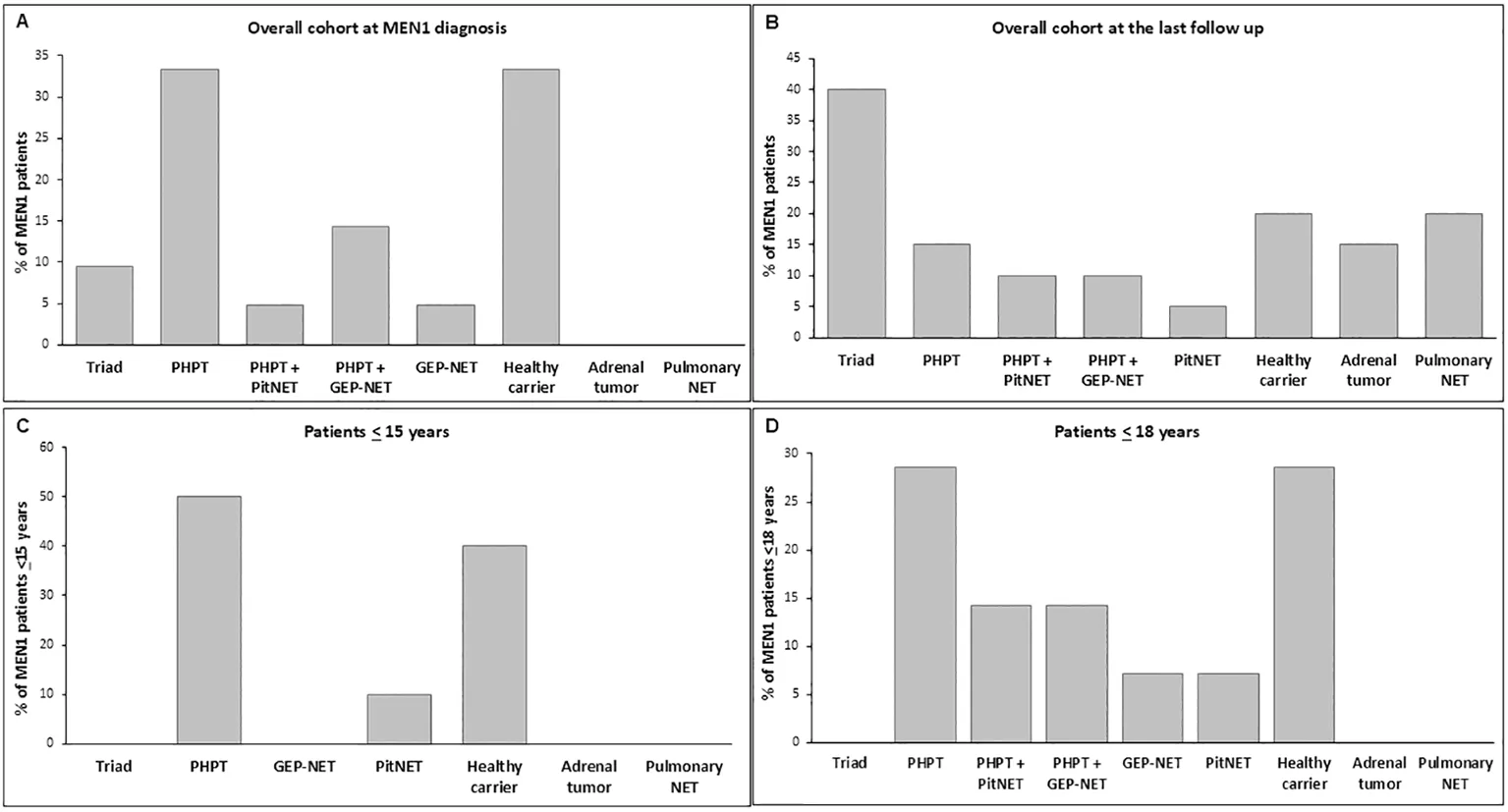
The distribution of MEN1-related manifestations at the diagnosis of MEN1 in the entire cohort **(A)**, at the last follow-up **(B)**, in patients ≤ 15 years old **(C)** and ≤ 18 years old **(D)**. It is presented as a percentage among patients who developed clinical features of the disease. PHPT, Primary hyperparathyroidism; GEP-NET, Gastroenteropancreatic neuroendocrine tumor; NET, Neuroendocrine tumor; PitNET, Pituitary neuroendocrine tumor.

At the time of initial evaluation, 7 (33%) patients were identified as healthy carriers of a *MEN1* P/LP variant without clinical manifestations, with a median age of 7 years (IQR 5-9). Two healthy carriers developed PHPT and a prolactin-secreting pituitary microadenoma at the ages of 12 and 8 years, respectively. The remaining 14 (67%) patients had already developed at least one major MEN1-related manifestation, and the distribution is reported in **Figure 1**. The median age of the first clinical manifestation was 17 years (IQR 15-19). In three patients, the genetic diagnosis of MEN1 was made following the onset of symptoms related to PHPT-related manifestations. Specifically, all three patients experienced recurrent episodes of nephrolithiasis.

In 19 (90%) patients, the genetic diagnosis was made as part of a screening program following the identification of MEN1 in a first-degree relative. In two cases, the affected individuals had a sporadic MEN1. One patient (#OS, **Table 1**) was found to carry a *de novo MEN1* variant, c.1087_1089delGAG. The proportion of patients who had symptomatic PHPT at the time of genetic diagnosis was similar between familial and apparently sporadic cases (3/19 vs. 0/2, p = 1.0). Follow-up data were available for 20 out of 21 patients (95%), with a median age of 27 years (IQR 15-28) at the most recent evaluation and a median follow-up duration of 9 years (IQR 6-11). Notably, four patients had a follow-up period exceeding 15 years, and eight patients were followed for more than 10 years. The distribution of MEN1-related manifestations is reported in **Figure 1**.

**Table 1:** Clinical and biochemical characteristics of MEN1-related primary hyperparathyroidism.

Patient ID	Age at diagnosis (years)/Sex	PHPT related-complications	Ionized calcium (mmol/L)	Total serum calcium (mg/dL)	PTH fold increase	Phosphate (mg/dL)	25(OH)D (ng/mL)	Urinary calcium (mg/24h)
AS	22/M	None	1.41	10.5	0.48	3.0	37.2	193
BD	15/M	Hypercalciuria Nephrolithiasis	1.38	10.3	0.72	3.6	39.0	425
CE	15/F	Hypercalciuria	1.42	10.2	1.38	2.7	33.0	405
CM2	14/F	None	1.49	11.5	0.95	3.2	22.3	234
CS	17/F	None	1.47	10.6	1.53	1.8	24.5	204
DCF	17/M	Hypercalciuria Reduced BMD	1.54	10.5	1.17	1.7	31.9	375
GA	15/M	Hypercalciuria Reduced BMD	1.51	11.7	0.89	3.1	37.0	448
GF	19/M	Hypercalciuria Nephrolithiasis Reduced BMD	1.65	12.3	1.57	2.7	21.9	404
IE	19/F	Nephrolithiasis	1.45	10.2	1.38	2.8	27.9	240
ME1	19/F	Hypercalciuria Reduced BMD	1.56	11.4	1.89	2.7	11.1	360
ME2	19/F	Hypercalciuria Reduced BMD	1.49	12.1	1.77	2.3	33.3	300
MS	21/F	Nephrolithiasis Reduced BMD	1.55	10.9	1.85	2.9	12.7	NA
OS	19/F	None	1.51	11.5	1.65	3.5	21.8	150
SFP	12/M	None	1.42	NA	1.15	NA	NA	NA
ZM	20/F	Hypercalciuria	1.38	10.2	0.78	2.6	22.9	430
Median (IQR)	18 (15-19)		1.49 (1.42-1.53)	11.4 (10.4-11.6)	1.38 (0.92-1.61)	2.7 (2.6-3.1)	24.5 (22-33)	368 (234-405)

F, female; M, male; PHPT, primary hyperparathyroidism; PTH, parathyroid hormone; 25(OH)D, 25-Hydroxyvitamin D; BMD, bone mineral density; NA, not available; IQR, interquartile range.

### Clinical and biochemical profile of PHPT

Fifteen of the 21 patients (71%) developed PHPT during follow-up, with a median age at diagnosis of 18 years (IQR 15–19, range 12–22). There was a female predominance, with 9 female patients (60%). When stratified by sex, the median age at presentation was 15 years (IQR 15-17) for males and 19 years (IQR 17–19) for females with no statistically significant difference (p = 0.087). Full clinical, biochemical, and urinary data of the patients are reported in **Table 1**. Biochemical comparison by sex showed higher 25(OH)D levels in males (33.4 ± 7.0 *vs* 23.3 ± 7.8 ng/mL; p = 0.033) whereas females tended to have a greater PTH fold increase compared with males (1.46 ± 0.39 *vs* 1.00 ± 0.38; p = 0.043).

Baseline assessment of PHPT-related complications revealed that 67% of patients (n=10) exhibited at least one complication (**Table 1**), distributed as follows: i) hypercalciuria (defined as urinary calcium excretion >4 mg/Kg/day) (26) in 8 of 14 (57%) patients with available 24-hour urinary calcium analysis; ii) silent nephrolithiasis in 4 of 14 (29%) patients with available renal ultrasound; iii) reduced bone mass in 6 of 14 (43%) patients with available DXA, specifically in 5 at one-third distal radius (median Z-score -2.7), in 3 at femoral neck and total hip (median Z-score -2.4 and -2.1, respectively) and in 2 at lumbar spine (median Z-score -2.4). No fragility fractures were reported. At diagnosis, bone turnover markers showed a median fold increase of 0.49 (IQR 0.23-1.10) for osteocalcin, 0.72 (IQR 0.48-1.59) for BSAP, and 0.54 (IQR 0.39-3.54) for S-CTX.

Follow-up data were available for 14 of 15 patients (93%).

Five (36%) patients underwent parathyroidectomy (PTX) at a median age of 22 years (IQR 19–25), due to progressive disease-related complications (**Table 2**). The final decision was based on shared discussion with the patient and with their parents, as appropriate. All patients underwent bilateral neck exploration as previously described (27). Briefly, less than subtotal parathyroidectomy (LSTP) consisted of excision of one or two pathological parathyroid glands, whereas subtotal parathyroidectomy (STP) involved removal of three or three and a half glands, leaving a small remnant of macroscopically normal parathyroid tissue *in situ*.

**Table 2:** Clinical, biochemical findings and management in patients with MEN1-related primary hyperparathyroidism at the last follow up.

Patient ID	Age at last follow up (years)	Management (Treatment)	Ionized calcium (mmol/L)	Total serumcalcium(mg/dL)	PTH fold increase	Phosphate (mg/dL)	25(OH)D (ng/mL)	Urinary calcium (mg/24h)	PHPT related-complications
Conservative management									
AS	28	Surveillance	1.32	10.4	0.59	3.5	27.0	207	None
BD	36	Surveillance	1.42	10.4	1.84	2.1	28.4	570	Hypercalciuria Reduced BMD
CE	29	Surveillance	1.39	10.2	1.73	2.6	33.4	322	Hypercalciuria
CM2	25	Surveillance	1.44	11.1	1.43	3.6	17.1	264	Hypercalciuria
CS	23	Surveillance	1.45	10.7	2.47	2.7	27.1	225	None
IE	27	Surveillance	1.44	10.6	1.23	2.5	33.9	280	Hypercalciuria
ME2^*^	28	Medical therapy (CINA 60 mg)	1.38	10.3	1.23	3.1	39.6	210	Hypercalciuria Reduced BMD
MS^*^	33	Medical therapy (CINA 60 mg)	1.40	11.3	1.80	2.9	33.1	408	Hypercalciuria Nephrolithiasis Reduced BMD
ZM	24	Surveillance	1.36	10.2	0.63	3.0	21.0	280	Hypercalciuria Reduced BMD
Median (IQR)	28 (25–29)		1.40 (1.38-1.44)	10.4 (10.3-10.7)	1.43 (1.23-1.8)	2.8 (2.6-3.0)	28.4 (27.1-33.4)	280 (225–322)	
Surgical management									
DCF	28	Cervical exploration (STP, 3.5 glands)	1.41	10.8	0.73	2.3	34.6	320	Hypercalciuria Reduced BMD
GA	35	Cervical exploration (STP, 3 glands)	1.42	10.8	1.42	1.8	27.8	595	Hypercalciuria Reduced BMD
GF^*^	40	Cervical exploration (LSTP, 2 glands) → Medical therapy (CINA 60 mg)	1.20	8.7	0.84	2.7	34.3	464	Hypercalciuria Reduced BMD
ME1^*^	28	Cervical exploration (STP, 3 glands) → Medical therapy (CINA 60 mg)	1.25	9.5	2.2	3.1	32.6	266	Hypercalciuria Reduced BMD
OS^*^	26	Cervical exploration (LSTP, 2.5 glands) → Chronic hypoPT (CaCO_3_ 1000 mgCalcitriol 0.5 mcg)	1.12	8.9	0.17	4.2	35.5	350	Hypercalciuria

*: Biochemical data are reported under ongoing daily therapy.

LSTP, less than subtotal parathyroidectomy; STP, subtotal parathyroidectomy; hypoPT, hypoparathyroidism; CINA, cinacalcet; CaCO_3_, calcium carbonate; PTH, parathyroid hormone; 25(OH)D, 25-Hydroxyvitamin D; BMD, bone mineral density; IQR, interquartile range.

SPT was performed in three cases, while one patient underwent removal of two glands, and another underwent two and a half PTX; in both instances, the remaining glands were preserved as they appeared macroscopically normal during surgical exploration. Histology showed parathyroid hyperplasia in all cases. Postoperatively, one patient (#OS) developed postoperative hypoparathyroidism. Persistent PHPT occurred in three patients, and one (#GA) experienced recurrence approximately 10 years after surgery. Two of these patients with persistent PHTP were treated with cinacalcet, while the others remained under active surveillance (**Table 2**).

Nine (64%) patients did not undergo surgery, and they were on active surveillance (**Table 2**). Adequate hydration was advised in all patients. Two patients were treated with cinacalcet 60 mg daily, resulting in a reduction in serum calcium during follow-up. The remaining 7 patients were under surveillance without any medical treatment. In these latter patients, during follow-up, ionized serum calcium concentrations significantly decreased compared to baseline (1.45 ± 0.06 *vs*. 1.40 ± 0.4 mmol/L; p = 0.032), whereas no significant change in PTH fold increase was observed (p = 0.12).

During the follow-up, bone turnover markers showed a median fold increase of 0.84 (IQR 0.51-1.07) for osteocalcin, 0.70 (IQR 0.59-1.22) for BSAP, and 0.78 (IQR 0.66-1.22) for S-CTX.

During the follow-up, seven patients (50%) were treated with cinacalcet according to EMA indications (28), with a median age at treatment initiation of 21 years; five of these patients started therapy between 19 and 21 years of age. Treatment was generally well tolerated; one patient discontinued therapy due to gastrointestinal intolerance, and dose reduction was required in another case (from 60 mg/day to 30 mg/day). At the last follow-up, four patients remained on cinacalcet therapy (60 mg/day).

### Clinical and biochemical profile of GEP-NET

Among the 21 subjects under surveillance, 10 (48%) developed a GEP-NET, identified by abdominal contrast-enhanced MRI. The sex distribution among affected subjects was 4 males (40%) and 6 females (60%), with no significant difference observed between the two groups (p = 0.67). The median age at diagnosis was 19.5 years (IQR 18–22), with the youngest patient diagnosed at 17 years. When stratified by sex, the median age at diagnosis was 23 years (21–24) in males and 19 years (18–20) in females. Considering the age threshold of 21 years, 33% (n=7) of patients developed a GEP-NET with a median age at diagnosis of 19 years (17.5–19.5), and most of these early-onset cases were female (86%).

Tumors were primarily located in the pancreas (90%), with one case exhibiting both pancreatic and duodenal ampullary involvement. In all but two patients, tumors were non-functioning; one patient was diagnosed with an insulinoma (#CS) and one (#CE) with a glucagonoma. The patient who developed the insulinoma had a prior diagnosis of non-functioning GEP-NET at the age of 17 and experienced neuroglycopenic symptoms at 20 years. No cases of gastrinoma or vasoactive intestinal peptide-secreting tumors were reported.

At diagnosis, 6 patients (60%) had a single lesion detected on imaging, whereas four had multiple lesions (≥2). The median size of the primary lesion was 1 cm (IQR 0.7–1.2), ranging from 0.5 to 2.1 cm, with half of the lesions measuring ≥1 cm.

Four patients (#BD, #CE, #CS, #MS) (40%) underwent surgical intervention, two due to hormone secretion (insulinoma and glucagonoma) and other two due to tumor progression based on lesion size. At the time of surgery, the median tumor size was 18.5 mm (IQR 16.8–22.5), and the mean preoperative growth was 1.22 ± 0.41 mm/year (median 1.10; range 0.88–1.80). Surgical procedures varied among the four cases as reported in **Table 3** and one patient (#MS) underwent surgery at another center. One patient (#CE) received somatostatin-analogues therapy for 3 years prior to the surgery.

**Table 3:** Clinical and instrumental characteristics of GEP-NETs.

Subject ID	Sex	Age at diagnosis of GEP-NETs (years)	Number of lesions (n)/max diameter (mm)*	Type of lesions	Age at last follow-up/ Surgery (years)	Number of lesions (n)/max diameter (mm)^*^	Growth rate (mm/year)^†^	Management	Grading/TNM
BD	M	22	1/8	NF	32	1/20	1.20	Surgery (lesion enucleation + lymph node sampling)	G2/pT1N0M0
CE	F	20	3/21	Glucagonoma	25	4/30	1.80	Surgery (left pancreatectomy + spleen preservation)	G2/pT1N0M0
CS	F	17	1/12	Insulinoma	21	2/16	1.00	Surgery (pancreatic-duodenectomy + tail lesion enucleation)	G2/pT1N0M0
DCF	M	17	2/14	NF	29	2/14	0.00	Surveillance	
GA	M	25	1/7	NF	36	2/10	0.27	Surveillance	
GF	M	24	2/6	NF	40	2/11	0.31	Surveillance	
MS	F	21	3/10	NF	29	6/17	0.88	Surgery (total pancreatic-duodenectomy)	G2/pT1N0M0
ME2	F	19	1/7	NF	31	1/7	0.00	Surveillance	
OS	F	19	1/5	NF	26	6/11	0.86	Surveillance	
ZM	M	18	1/11	NF	25	2/11	0.00	Surveillance	

F, female; M, male; GEP-NET, gastroenteropancreatic neuroendocrine tumor; NF, nonfunctioning. * For surgically treated patients, the number of lesions/and maximum tumor diameter refer to the surgical time; † For surgically treated patients, the annual growth rate was estimated based on change in maximum tumor diameter and the age at the time of surgery.

Histological examination confirmed well-differentiated grade G2 NETs in all cases (Ki-67 proliferation 2.7-4%). According to 2017 WHO TNM staging (29), all tumors were classified as pT1N0. Immunohistochemistry was positive for chromogranin and synaptophysin in all cases, with hormone-specific staining consistent with the clinical phenotype.

Postoperative acute complications included peripancreatic fluid collections in three patients (two requiring drainage, Clavien-Dindo grade IIIa) (30) and low-output pancreatic fistulas, classified as biochemical leaks according to the International Study Group of Pancreatic Surgery (ISGPS) definition (31), in two. One patient developed postsurgical diabetes after total pancreatectomy, achieving good glycemic control with an automated insulin delivery system. Following surgery, patients underwent serial biochemical and radiological follow-up with MRI. After a median follow-up of 5 years, only one patient showed a stable hypervascular pancreatic focus suggestive of persistent disease, and no metastatic progression was observed.

The remaining six patients were managed with regular gadolinium-enhanced MRI imaging and biochemical surveillance. After a median follow-up of 11 years, the mean NET growth rate from diagnosis to last follow-up was 0.23 ± 0.33 mm/year (median 0.12; range 0–0.86) (**Table 3**).

### Clinical and biochemical profile of PitNET

Eleven of 21 patients (52%) developed a PitNET, with a sex distribution of seven females (63.6%) and four males (36.4%) (**Table 4**). The median age at diagnosis was 21 years (IQR 19–23), ranging from 8 to 38 years. In only one case was PitNET, the first manifestation of MEN1. In eight patients, the adenoma was diagnosed at or before the age of 21, accounting for 38% (n=8) of the entire cohort. Among these early-onset cases, the median age at diagnosis was 19 years (17.8-21) with a female prevalence (63%, n=5) but no gender difference emerges (p = 0.20). The median adenoma size of the entire cohort at diagnosis was 4 mm (IQR 3-5.5).

**Table 4:** Characteristics of patients with PitNET.

Subject ID	Sex	Age of diagnosis of PitNET (year)	Largest diameter at diagnosis (mm)	Hormonal secretion	Management	Age at the last follow-up (years)	Largest diameter at the last follow-up (mm)
BD	M	17	6	Absent	Surveillance	36	6
CE	F	24	1	Absent	Surveillance	29	7
CM2	F	18	6	Absent	Surveillance	25	6
DCF	M	27	3	Absent	Surveillance	NA^*^	NA^*^
GF	M	38	5	Absent	Surveillance	40	5
ME1	F	19	4	Absent	Surveillance	28	5
ME2	F	19	3	Absent	Surveillance	28	4
MS	F	21	5	Absent	Surveillance	33	5
RG	M	8	9	Prolactin	Medical therapy (Cabergolin)	11	5
OS	F	21	3	Absent	Surveillance	26	3
ZM	F	21	3	Absent	Surveillance	NA^*^	NA^*^

F, female; M, male; NA, not available; PitNET, pituitary neuroendocrine tumor; * Data are not available because the pituitary MRI has not yet been performed.

**Figure 2:**
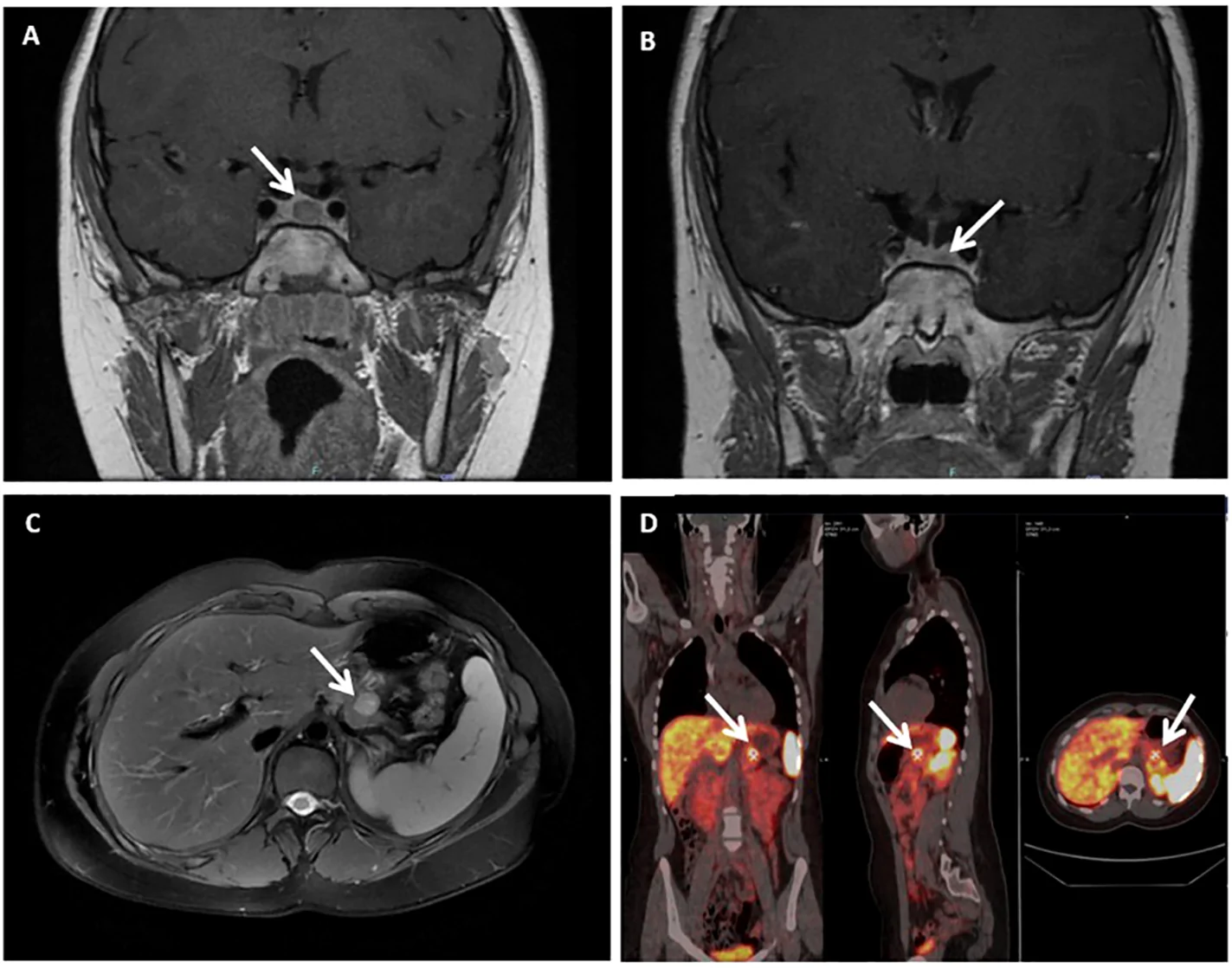
**(A)** T1-weighted coronal scan in Pituitary MRI of patient RG at diagnosis showing a prolactinoma localized in the anterior pituitary gland at baseline (arrow). **(B)** Follow-up pituitary MRI after 36 months of dopamine agonist therapy of the same patient, demonstrating significant tumor shrinkage with marked reduction in lesion size and at its original site, only a faint area of contrast hypointensity remains (arrow). **(C)** Pancreatic MRI of patient CS revealing a focal hypervascular lesion (16 mm) consistent with insulinoma (arrow). **(D)** Functional imaging (68Ga-DOTA PET/CT) of the same patient confirming increased tracer uptake in the pancreatic lesion (arrow), consistent with a well-differentiated neuroendocrine tumor.

All PitNETs were non-functioning except for one. In one patient (#RG), an 8-year-old boy, was diagnosed with 9 mm prolactinoma (prolactin 147 mcg/L; reference range 2-13) (**Figure 2**). This patient started treatment with cabergoline (0.25 mg twice weekly), resulting in marked tumor shrinkage and complete radiological resolution after 36 months (**Figure 2**). In the overall PitNET cohort, the median follow-up was 5 years (IQR 2.5-9).

Among the nine patients with available adenoma size data at the last follow-up, the median lesion diameter was 5 mm (IQR 5–6). Tumor size remained stable in most cases, with only slight enlargement observed in two patients. In the eight patients with serial imaging, excluding the patient treated with cabergoline, the mean adenoma growth rate was 0.17 ± 0.42 mm/year (range 0–1.2 mm/year). Paired analysis showed no significant difference between adenoma size at diagnosis and at follow-up (p = 0.181) (**Figure 2**; **Table 4**).

### Adrenal tumors

Adrenal involvement was observed in three of 21 patients (14%). All lesions were detected during routine MEN1 surveillance by abdominal imaging (ultrasonography, MRI and/or CT). Two patients presented with adrenal adenomas and one with bilateral adrenal hyperplasia.

Both adenomas were non-functioning and were diagnosed at 19 (#DCF) and 29 (#ME1) years of age, with initial sizes of 20 mm and 10 mm, respectively. One patient (#DCF) showed progressive tumor growth over 10 years, reaching 62 mm, with heterogeneous radiologic features prompting unilateral adrenalectomy. Histology revealed an oncocytic adrenocortical tumor. In the other case (#ME1), lesion size remained stable during two years of follow-up, although diffuse hyperplasia of the contralateral adrenal gland was noted.

The patient with bilateral adrenal hyperplasia (#MS) was diagnosed at 21 years and followed for 14 years, without morphological progression or evidence of hormonal hypersecretion.

### Other endocrine and non-endocrine manifestations

Pulmonary NETs were detected in four patients (19%) during follow-up, all over the age of 21, with a median age at diagnosis of 28.5 years (IQR 27.5–30). In two patients, bilateral subcentimetric nodules were observed; the remaining two patients had a solitary nodule. In one case, the surgical resection confirmed an atypical carcinoid tumor (G2, pT1a) with a Ki-67 index of 3% and low mitotic activity. During a median follow-up of 2.5 years, no significant changes in nodule size were observed in any patient.

In a separate case (#MS), an incidental endotracheal radiopaque lesion was detected on CT scan in a 34-year-old asymptomatic patient and successfully removed endoscopically. Histology revealed a hamartochondroma, with no recurrence at one-year follow-up.

Cutaneous manifestations, specifically angiofibromas, were observed in seven patients (33%), with a median age at presentation of 23 years (IQR 21.5–27; range 9–31). These lesions were primarily localized to the face (nasal area and lower lip) and chest. Lipomas, both cutaneous and visceral, were identified in 4 patients (19%), predominantly affecting the thoracic and abdominal regions with a median age of 22 years (IQR 21-24). Patients exhibited between one and five lipomas. Surgical excision was performed in three cases, with histological diagnoses of lipoma in two and lipoblastoma in one. Two patients experienced local recurrence following excision after 10 years.

### MEN1 genetic analysis

**Figure 3:**
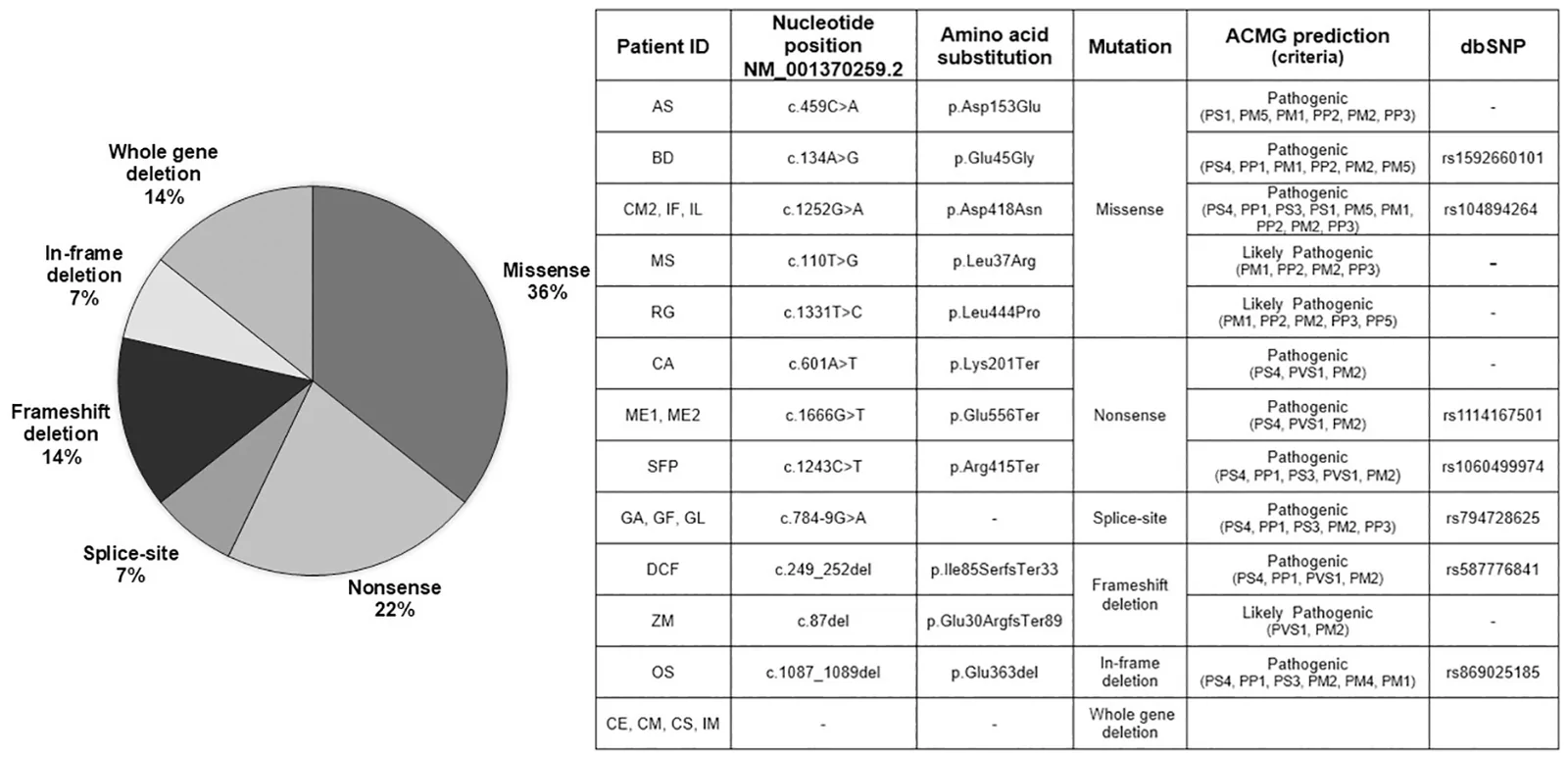
*MEN1* germline variants identified in the study cohort. Variants are grouped by genetic variant type (missense, nonsense, splice-site, frameshift and whole-gene deletion).

Genetic testing was performed in all patients and identified a *MEN1* P/LP variant in all cases. Most of the *MEN1* variants had been previously reported, including two previously reported by our group (2, 32). Twelve distinct *MEN1* variants were identified, predominantly involving exon 2 (42%) and exon 9 (25%). According to the ACMG classification, nine variants were classified as pathogenic and three as likely pathogenic. In addition, a germline whole-gene *MEN1* deletion identified by MLPA was detected in four patients belonging to two unrelated families (**Figure 3**). Overall, 58% of variants were predicted as loss-of-function alterations (frameshift, nonsense, canonical splice-site variants, or deletions).

## Discussion

MEN1 typically manifests in adulthood, increasing evidence highlights the early onset of the disease, even in pediatric and adolescent populations (13–15).

In this single-center cohort, disease manifestations were frequently detected early in life. The median age at genetic diagnosis was 15 years, and approximately one third of patients were identified as presymptomatic carriers through familial screening. This finding underscores the role of genetic testing and structured surveillance in at-risk families (4, 33), but it also indicates that our cohort reflects early diagnosis through active screening as much as intrinsically early disease onset. Therefore, the young age at diagnosis observed in our cohort likely resulted from both the disease predisposition of MEN1 manifestations to occur during adolescence and the implementation of proactive familial screening.

The earliest and most frequent clinical manifestation was PHPT (**~**70%), consistent with previous studies (16). Recurrent nephrolithiasis preceded MEN1 diagnosis in a subset of patients, suggesting MEN1 as an important differential diagnosis in early-onset nephrolithiasis. Over long-term follow-up (median 9 years), MEN1 exhibited a progressive pattern, characterized by an increasing number of manifestations and a higher proportion of patients satisfying the criteria for the classic triad (**~**40%), although this observation should be interpreted in light of the screening-driven nature of case ascertainment and the variable age at last follow-up. Consistent with previous pediatric and young adult MEN1 series, PHPT frequently developed before the age of 20, with cases observed as early as 12 years (4, 18, 34, 35). Although the biochemical phenotype was sometimes mild, the clinical impact was substantial: two-thirds of patients presented with at least one complication, mainly hypercalciuria and silent nephrolithiasis. These complications may occur even in the setting of mild hypercalcemia and significantly influence management decisions, underscoring the importance of systematic renal evaluation at diagnosis (36, 37). Skeletal involvement was also frequent predominantly at cortical sites, such as the one-third distal radius. This pattern is consistent with the preferential cortical bone involvement characteristic of PHPT (38, 39). DXA interpretation in adolescents must account for growth and pubertal influences, Similarly, bone turnover markers must be interpreted in the context of physiological pubertal elevation (40).

PTX remains the only curative therapy for MEN1-related PHPT. However, surgical management in adolescents is complex due to multiglandular involvement and the well-documented risk of persistence or recurrence (41, 42). In our series, surgery was performed in a minority of patients, primarily in those with progressive or clinically significant complications. Persistent or recurrent PHPT was observed in several cases, consistent with previous reports, including the series by Shariq et al., in which, after a median postoperative follow-up of 62 months, LSPT was associated with significantly worse disease-free outcomes, with persistent or recurrent disease reported in >85% of patients, compared with >20% after SPT and 0% after total PTX (17). Some authors have suggested that, in selected cases, particularly in very young patients, a less extensive initial procedure may be considered and the most recent MEN1 guidelines indicate that unilateral clearance PTX may be considered as the initial operation in selected children or adolescents with MEN1-related PHPT (6, 41). Overall, these considerations underscore the challenge of achieving durable remission while minimizing the risk of permanent hypoparathyroidism, supporting careful and individualized surgical decision-making in young patients.

Among patients managed conservatively, a modest but significant reduction in calcium concentration was observed over time. This may partly reflect structured follow-up and optimized supportive measures, including adequate hydration and adequate calcium intake, as suggested by international guidelines (43). Of note, low dietary calcium may exacerbate parathyroid hypersecretion. Nevertheless, the small number of patients included, and the observational nature of the analysis preclude definitive conclusions regarding the natural course of the disease or the effectiveness of conservative management.

Cinacalcet, an allosteric modulator of the calcium-sensing receptor, was used in patients with persistent disease after surgery or in those with asymptomatic disease, but with an increase in serum calcium (>11.2 mg/dL). All treated patients were over 19 years of age. The treatment was generally well tolerated, with gastrointestinal side effects requiring dose adjustment in a minority. While long-term safety data in pediatric populations remain limited, emerging multicenter experiences provide preliminary reassurance regarding its use in carefully selected young patients (44). Decisions regarding cinacalcet initiation should therefore balance the benefits of biochemical control against the still limited long-term evidence in this age group.

GEP-NETs were detected in approximately one-third of the patients, a prevalence within the range reported in pediatric and adolescent series (9-40%). Variability across studies likely reflects differences in age thresholds, follow-up duration, and imaging strategies. In our cohort, abdominal MRI represented the main screening modality, while EUS and functional imaging were used selectively according to lesion characteristics and clinical indication; therefore, detection of very small lesions may have varied over time as imaging tools evolved. While CT and MRI are routinely employed, EUS provides greater sensitivity for small lesions but is invasive and less consistently used in younger populations (13, 17, 18). In our series, GEP-NETs were diagnosed at a median age of 19 years and were asymptomatic at detection, highlighting the contribution of surveillance programs to lesion identification before clinical presentation. Lesions were predominantly pancreatic and mainly solitary, consistent with previously described pediatric patterns (13). The distribution of clinically relevant nonfunctioning pancreatic NETs in our cohort aligns with data showing that such lesions are uncommon before 18 years of age but increase in frequency during late adolescence (5, 45). Only two tumors were functioning, one insulinoma and one glucagonoma. Insulinomas have been reported in 10–25% of juvenile MEN1 patients and may arise after a prior diagnosis of a non-functioning GEP-NET, suggesting dynamic tumor evolution (5, 16, 46, 47). No gastrinomas were identified, in line with most pediatric series, although early cases have been described (14, 18). Importantly, no patient developed metastatic disease during follow-up, consistent with observations from large registries in young MEN1 patients. Nevertheless, metastatic cases in adolescence have been reported, suggesting the need for careful longitudinal monitoring (5, 14, 17). Surgical intervention was required in fewer than 40% of the cases, primarily for tumors exceeding 20 mm or demonstrating significant growth, in accordance with current guideline-based thresholds (48). Tailored surgical approaches, guided by tumor location and multifocality, and supported by multimodal imaging (including EUS, MRI, and functional imaging), resulted in favorable outcomes (46, 49–51). Among patients managed conservatively, tumor growth was generally slow over long-term follow-up. Although some increase in lesion size was observed, the overall behavior was indolent. These findings reinforce that many non-functioning GEP-NETs detected in adolescence follow a relatively stable course, while emphasizing the importance of structured surveillance to identify the subset of tumors that may progress. Collectively, our data suggest that early and individualized pancreatic screening, beginning in adolescence, may be appropiate while balancing diagnostic sensitivity against procedural burden (13, 17).

PitNETs were identified in more than half of the patients, with some diagnoses occurring in childhood, consistent with reports of very early pituitary involvement in MEN1 (14). Most were nonfunctioning microadenomas that remained stable over time, in agreement with the literature (16, 52, 53). This distinction between structural detection and clinically significant disease is particularly relevant in young MEN1 patients: early identification through surveillance often reveals small, asymptomatic lesions that may never require intervention. Only one prolactinoma was observed and responded well to dopamine agonist therapy. Although rare aggressive pituitary presentations have been described in pediatric MEN1 (52), our findings suggest that, when detected through surveillance and monitored appropriately, pituitary disease in this age group is often indolent and manageable conservatively (54) Early surveillance may facilitate identification of pituitary lesions at a stage where conservative management remains feasible (48, 55, 56).

Adrenal involvement was uncommon and predominantly nonfunctioning, consistent with the literature data (57). Although most lesions remained stable, one adenoma showed substantial interval growth and required adrenalectomy, illustrating that surveillance may be essential even for lesions that are initially asymptomatic. Although rare, malignant adrenal tumors have been reported in pediatric MEN1, including adrenocortical carcinoma with clinical signs of hyperandrogenism in young girls (14, 58), and interval growth of pediatric adrenal lesions has also been described (17). Overall, most adrenal findings are indolent but require individualized long-term monitoring (14, 57).

Cutaneous manifestations, particularly angiofibromas (~one-third) and lipomas, were frequent and may serve as early clinical clues to MEN1 diagnosis. This is supported by our data in MEN1 patients showing higher rates of facial angiofibromas/lipomas in familial MEN1 compared to sporadic MEN1 and in patients with a positive *MEN1* variant compared to *MEN1* variant-negative (32). Pulmonary nodules were detected after age 21 and were mostly small and stable, although one patient had an atypical pulmonary carcinoid, a recognized (albeit uncommon) MEN1 manifestation (14) and reported in other series (16, 59).

This study provides single-center data on MEN1 diagnosed in children, adolescents, and young adults (≤21 years), a population for which evidence remains comparatively limited. A key strength is the longitudinal design with high follow-up completeness and a substantial follow-up duration (median 9 years; several patients followed for >10–15 years), allowing description of the disease evolution and treatment outcomes over time. Finally, the manuscript captures a broad MEN1 phenotype, including not only the classic triad (PHPT, GEP-NETs, PitNETs) but also adrenal, cutaneous and thoracic manifestations, offering a more comprehensive picture of MEN1 morbidity in early life.

The main limitations stem from the retrospective, single-center design, which may introduce referral/ascertainment bias and limit external generalizability. The small sample size (n=21) intrinsically restricts the robustness of prevalence estimates, limits statistical power, especially for subgroup analyses (e.g., sex-stratified comparisons) and requires cautious interpretation of descriptive comparisons. Moreover, the long enrolment window (2001–2024) implies potential temporal heterogeneity in surveillance protocols and diagnostic tools. Although the overall surveillance approach was based on guideline recommendations, the evolution of MRI technology and selective use of complementary imaging modalities, may have increased the detection of small GEP-NETs and PitNETs over time.

In this single-center cohort of genetically confirmed MEN1 patients diagnosed at ≤21 years, early disease expression was common and clinically meaningful. PHPT was the earliest and most frequent manifestation, often associated with renal and skeletal involvement despite occasionally mild biochemical abnormalities. GEP-NETs and pitNETs were also common in late adolescence and young adulthood, although many lesions were non-functioning, small, and radiologically detected during surveillance. Overall, our data suggest the importance of early genetic testing in at-risk families and age-adapted, multidisciplinary surveillance strategies that balance timely detection of morbidity with minimization of procedural burden in young individuals.

## Data Availability

The original contributions presented in the study are publicly available. The raw data supporting the conclusions of this article will be available from the corresponding author on reasonable request.
