# Supplementary material for: Clinical features of MEN1 in children, adolescents, and young adults: a single-center study
Source: Front Endocrinol (Lausanne). 2026 Aug 17;17:1886831. doi: 10.3389/fendo.2026.1886831 (PMC13526606; doi:10.3389/fendo.2026.1886831)
Supplement: Supplementary file 2 [file Table2.docx]

**Table 2**: Age and Sex-specific reference range for S-CTX (pg/mL)

| **Age Group** | **Males** | **Females** |
| --- | --- | --- |
| **0-2** | 244.7 - 1420.7 | 317.6 - 1245.1 |
| **3-4** | 337.1 - 1776.8 | 383.1 - 1553.0 |
| **5-6** | 301.0 - 1795.4 | 467.9 - 2112.6 |
| **7-8** | 428.2 - 2440.7 | 285.6 - 2179.2 |
| **9-10** | 336.1 - 2734.3 | 484.7 - 2834.1 |
| **11-12** | 277.4 - 3009.2 | 485.6 - 3667.1 |
| **13-14** | 218.1 - 4779.1 | 60.2 - 2443.0 |
| **15-16** | 153.4 - 3443.3 | 116.1 - 2039.8 |
| **17-18** | 82.2 - 2929.4 | 36.3 - 1104.6 |
| **19-20** | 95.1 - 1335.2 | 25.6 - 1116.3 |
