# Supplementary material for: Clinical features of MEN1 in children, adolescents, and young adults: a single-center study
Source: Front Endocrinol (Lausanne). 2026 Aug 17;17:1886831. doi: 10.3389/fendo.2026.1886831 (PMC13526606; doi:10.3389/fendo.2026.1886831)
Supplement: Supplementary file 3 [file Table1.docx]

**Table 1**: Age and Sex-specific reference range for bone-specific alkaline phosphatase.
